# Molecular Characterization of the First Partitivirus from a Causal Agent of *Salvia miltiorrhiza* Dry Rot

**DOI:** 10.3390/jof10030179

**Published:** 2024-02-27

**Authors:** Luyang Song, Rongrong Zhong, Zhengzhe Guan, Lina Huang, Ganlin Wang, Zhimin Yang, Ke Shao, Yanhong Qin, Caiyi Wen, Ying Zhao, Fei Wang

**Affiliations:** 1 College of Plant Protection, Henan Agricultural University, Zhengzhou 450046, China; lyang.s@webmail.hzau.edu.cn (L.S.); wencaiyi@henau.edu.cn (C.W.); 2 The Agriculture and Rural Development Service Center of Kaifeng City, Kaifeng 475499, China; 3 Inner Mongolia Academy of Science and Technology, Hohhot 010020, China; 4 Institute of Plant Protection, Henan Academy of Agricultural Sciences, Zhengzhou 450002, China

**Keywords:** partitivirus, *Fusarium oxysporum*, *Salvia miltiorrhiza* dry rot, hypovirulence

## Abstract

Root rot as a result of *Salvia miltiorrhiza* is a common root disease caused by *Fusarium* spp., which has become one of the main diseases affecting the production of *S. miltiorrhiza*. Currently, several hypovirulence-related mycoviruses have been identified in many phytopathogenic fungi, including *Fusarium* spp., which show potential as biological controls. In this study, we report a new mycovirus, Fusarium oxysporum partitivirus 1 (FoPV1), isolated from *F. oxysporum* strain FCR51, which is a causal agent of *S. miltiorrhiza* dry rot. The FoPV1 genome contains two double-stranded RNA segments (dsRNA1 and dsRNA2). The size of dsRNA1 is 1773 bp, and it encodes a putative RNA-dependent RNA polymerase (RdRp). The dsRNA2 is 1570 bp in length, encoding a putative capsid protein (CP). Multiple sequence alignments and phylogenetic analyses based on the amino acid sequences of the RdRp and the CP proteins indicated that FoPV1 appears to be a new member of the family *Partitiviridae* that is related to members of the genus *Gammapartitivirus*. Pathogenicity assay showed that FoPV1 confers hypervirulence to its host, *F. oxysporum*. This is the first report of a partitivirus infecting *F. oxysporum* and the first hypovirulence-related mycovirus from the causal agent of *S. miltiorrhiza* dry rot.

## 1. Introduction

Mycoviruses are a group of viruses that infect almost all major filamentous fungi, oomycetes, and yeasts [1,2]. As a result of high-throughput virome analyses of various fungi, more and more mycoviral sequences have been sequenced and logged in the National Center for Biotechnology Information (NCBI) database [3]; these have been grouped into about 19 families by the International Committee on Taxonomy of Viruses (ICTV; https://ictv.global/taxonomy, accessed on 20 January 2024). Most mycoviruses are divided into single-stranded DNA (ssDNA), positive single-stranded RNA (+ssRNA), double-stranded RNA (dsRNA), and negative single-stranded RNA (-ssRNA) as the types of genomes [1,2,4]. Most dsRNA mycoviruses are classified into nine families (*Amalgaviridae*, *Chrysoviridae*, *Curvulaviridae*, *Megabirnaviridae*, *Partitiviridae*, *Polymycoviridae*, *Quadriviridae*, *Spinareoviridae*, *Totiviridae*) and one unassigned genus (*Botybirnavirus*) [5].

Viruses in the family *Partitiviridae* have been reported from fungi, plants, and protozoa [6,7]. For the fungi host, the members of the family *Partitiviridae* have been reported from phytopathogenic fungi (*Aspergillus fumigatus*, *Botrytis cinerea*, *Cytospora sacchari*, *Rhizoctonia solani*, *Ustilaginoidea virens*, etc.), oomycete (*Pythium nunn*), biological control fungi (*Trichoderma harzianum*), and even human pathogenic fungi (*Talaromyces marneffei*) [8,9,10,11,12,13,14,15,16,17,18,19,20,21]. In general, the mycoviruses in this family contain two dsRNA segments which have a 1.4 to 2.4 kb genome. Each of the dsRNA segments consists of one open reading frame (ORF), encoding the RNA-dependent RNA polymerase (RdRp) and the capsid protein (CP), respectively [6,7]. According to evolutionary analysis, viruses in the *Partitiviridae* family are divided into five recognized and two proposed genera: *Alphapartitivirus*, *Betapartitivirus*, *Cryspovirus*, *Deltapartitivirus*, *Gammapartitivirus*, *Epsilonpartitivirus,* and *Zetapartitivirus* [6,18,22].

Mycovirus infection usually causes no associated symptoms to the fungal hosts [1,23]. Some mycoviruses from pathogenic fungi could reduce the virulence of their fungal hosts to the plants, and this property is termed hypovirulence [1,23]. Many hypovirulence-associated mycoviruses have been reported in various plant pathogenic fungi such as *Botrytis cinerea*, *Rhizoctonia solani*, *Colletotrichum liriopes*, etc. [16,24,25,26]. The members of the partitivirus also have some hypovirulence-associated mycoviruses, like Sclerotinia sclerotiorum partitivirus 1 (SsPV1/WF-1), Colletotrichum liriopes partitivirus 1 (ClPV1), Botrytis cinerea partitivirus 2 (BcPV2), and Colletotrichum alienum partitivirus 1 (CaPV1) [16,17,18,27]. Some partitiviruses induced host hypovirulence by co-infecting with other mycoviruses, like Rosellinia necatrix partitivirus 1 (RnPV1) and Rosellinia necatrix partitivirus 10 (RnPV10) [20,28]. Moreover, Talaromyces marneffei partitivirus-1 (TmPV1) can enhance the virulence of its fungal host [21].

*F. oxysporum* is an important plant pathogenic fungus that is generally recognized as causing vascular wilt in various crop species [29]. In recent years, it has been shown that *F. oxysporum* could infect many crop roots, leading to root rot disease and causing serious losses to agricultural production [30,31]. Using hypovirulence-associated mycoviruses to control plant diseases provides a new scheme for plant disease control: CHV1 has been successfully used to control chestnut blight [32], and SsHADV1 is used to control the disease caused by *S. sclerotiorum* [33,34]. Some mycoviruses have been reported in *F. oxysporum* and only three of them (Fusarium oxysporum alternavirus 1 (FoAV1), Fusarium oxysporum f. sp. dianthi virus 1 (FodV1), and Fusarium oxysporum ourmia-like virus 1 (FoOuLV1)) are known to cause hypovirulence [35,36,37]. To our knowledge, all the mycoviruses reported in *F. oxysporum* were isolated from those causing vascular wilt diseases, while not isolated from those causing from root rot disease.

*S. miltiorrhiza* is an important Chinese medicinal material using dried roots and rhizomes as medicine, which has the effects of promoting blood circulation, regulating menstruation, sedating and tranquilizing, reducing swelling, and relieving pain [38]. Due to the single planting mode and the reasons for repeated cropping, the root rot of *S. miltiorrhiza* caused by *Fusarium* spp., which has become one of the main diseases, seriously threatened the safety and healthy development of *S. miltiorrhiza* industry [31]. Biocontrol agents have proven to be effective tools for controlling root rot diseases [39,40]. As more and more hypovirulence-associated mycoviruses were reported to control plant fungal diseases, a new idea to control root rot disease with hypovirulence-associated mycoviruses was made available.

In this study, we identified and characterized a novel hypovirulence-relevant partitivirus, namely Fusarium oxysporum partitivirus 1 (FoPV1), in *F. oxysporum* strain FCR51 isolated from diseased roots infected with *S. miltiorrhiza* dry rot in Henan province of China. Sequencing and phylogenetic analyses indicated that it is a novel member of the family *Partitiviridae*. Moreover, we verified the hypovirulence trait of FoPV1 on the *F. oxysporum* strains and evaluated its biocontrol potential for the root rot of *S. miltiorrhiza.*

## 2. Materials and Methods

### 2.1. Strains and Culture Conditions

Three *F. oxysporum* strains (FCR51, FCR24, FCR51-VF, DS42-2) were used in this study. The FCR51 and FCR24 strains were isolated from a diseased *S. miltiorrhiza* in Fangcheng County, Henan Province, China, in 2022. Virus-free FCR51 (FCR51-VF) is a detoxified strain of FCR51 obtained by hyphal tip detoxification. DS42-2 is a pathogen of *S. miltiorrhiza* root rot disease as control. All strains were stored in an ultra-low temperature refrigerator (−80 °C) with a 25% glycerol concentration, and cultured on potato dextrose agar (PDA) medium in the dark at 28 °C. Mycelium was cultured on PDA plates covered with cellophane membrane at 28 °C in the dark for 4–5 days for dsRNA extraction.

### 2.2. Extraction and Purification of DsRNA

The method of extracting dsRNA was as described previously [35]. The strain was cultured on PDA medium covered with cellophane membranes in the dark for 4–5 days, and 1–2 g of fresh mycelium was harvested. Isolation of dsRNA was performed by selective absorption of cellulose powder CF-11 (Sigma-Aldrich, St. Louis, MO, USA), while nucleic acid co-precipitating agent was added to increase the yield of dsRNA. The dsRNA was treated with RNase-free DNase I and S1 nuclease (Takara, Dalian, China), and the obtained segments were subjected to 1% agarose gel electrophoresis and filtered with ethidium bromide, while using a gel recording and image analysis system (InGenius LhR, Syngene, Cambridge, UK) for visualization.

### 2.3. Synthesis, Cloning and Sequencing of cDNA

The purified dsRNA was used as a template for cDNA synthesis. The synthesis and cloning of cDNA were performed as previously described [41]. The cDNA library was constructed using TransScript One-Step gDNA Removal and cDNA Synthesis SuperMix (TransGen Biotech, Beijing, China) according to the instructions. Initial clone sequences were obtained by RT-PCR amplification using random primers (RACE3RT). Partial gaps between the initial sequences were filled in by RT-PCR, with specific primer sequences designed based on the obtained sequences. The 5’ and 3’ terminal sequence cloning were carried out using T4 RNA ligase to ligate the anchor primer PC3-T7 loop to dsRNA and used it for RT reaction. The primer PC2 was designed based on the corresponding sequence of the PC3-T7 loop, and specific primers were designed based on the sequence of the proximal regions; these primers were used to amplify terminal sequences. All PCR products were cloned into the pMD19-T vector (Takara, Dalian, China), then transformed into *E. coli* DH5α (TaKaRa) and sequenced. Each nucleotide of the full-length cDNA was sequenced at least three times to obtain a high-quality consensus sequence. Supplementary Table S1 shows all primers for cDNA cloning and sequencing.

### 2.4. Sequence and Phylogenetic Analysis

The prediction of open reading frames (ORFs) and conserved domains was carried out via ORF finder and CD searches on the NCBI website (http://www.ncbi.nlm.nih.gov, accessed on 22 January 2024). Multiple sequence alignments were performed; moreover, multi-sequence analysis with members of other partitivirus were performed using the CLUSTALX (2.0) program [42]. The phylogenetic tree was constructed using MEGA11 software and generated through the maximum-likelihood (ML) method with 1000 bootstrap replicates [43]. The Phylogenetic tree was enhanced using ITOL (https://itol.embl.de/, accessed on 22 January 2024).

### 2.5. Acquisition of Virus-Free Mycoviruses

Mycoviruses proliferate and spread as the hyphal cells divide and grow, but different viruses may proliferate slower than the division of fungal cells; thus, the virus content in the tender tips of the hyphae may be low or even virus-free. Ribavirin can inhibit the replication of viruses and thus inhibit their spread. The FCR51 strain was cultured in PDA containing ribavirin (100 μg/mL) for 2–3 days, and the tips of the hyphae were picked and repeated 2–3 times. The virus-free mycoviruses can be detected by extracting dsRNA and combining it with RT-PCR detection.

### 2.6. RNA Extraction and RT-PCR Detection

The strains were cultured on PDA plates covered with cellophane membranes in the dark for 4–5 days, and then fresh mycelium was collected and ground into powder with liquid nitrogen for the extraction of total RNA. Total RNA was extracted according to the instructions of RNA reagent (TransGen Biotech, Beijing, China).

HiScript III 1st Strand cDNA Synthesis Kit (+gDNA wiper) (Vazyme Biotech Co., Ltd., Nanjing, China) was used to synthesize first-strand cDNAs for RT-PCR detection, and then specific primers designed based on the sequences of FoPV1 (listed in Supplementary Table S1) for PCR amplification, for detecting the virus-transmitted strains. The product amplified by RT-PCR was observed by electrophoresis in 1.0% agarose gel.

### 2.7. Assay of Biological Properties of Mycovirus-Infected and Mycovirus-Free Strains

The effect of mycoviruses is determined by measuring biological characteristics such as colony morphology, growth rate, conidia production, and virulence. The growth of the strains was observed and photographed at 1 day post-inoculation (dpi), 3 dpi, and 5 dpi. To obtain the growth rate, all active strains were cultured on fresh PDA plates and the colony diameter was measured at 3 dpi and 5 dpi. The pathogenicity of the strains was determined by pot experiments. All assays were repeated three times. Experimental data were analyzed by the Prism 8.0.2 software. Treatment means were compared using the least significant difference test with *p* = 0.05.

## 3. Results

### 3.1. DsRNA Segments in F. oxysporum Strains

**Figure 1:**
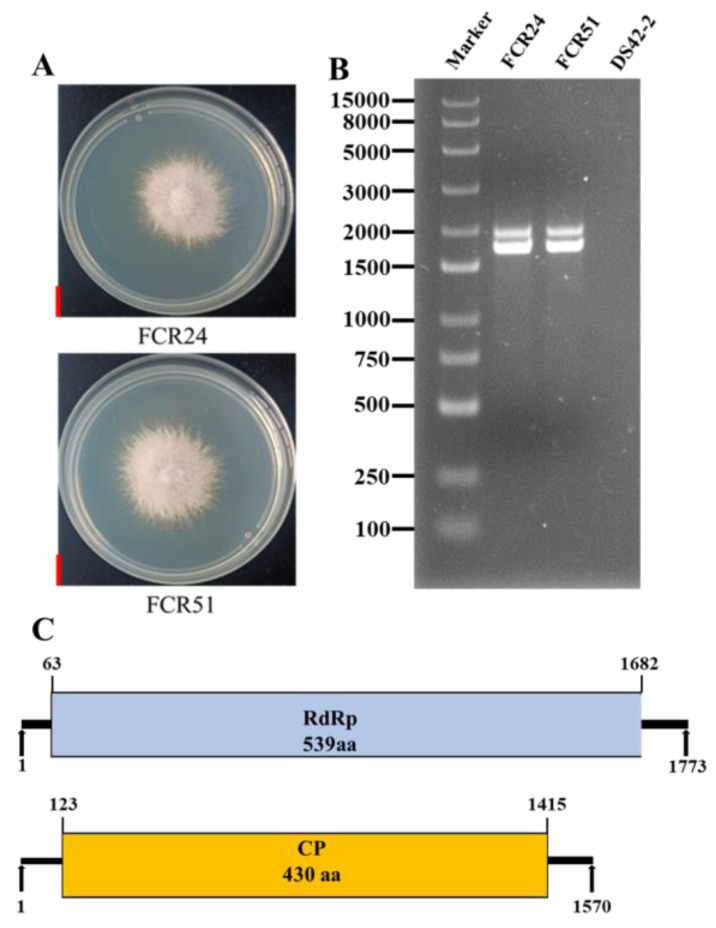
(**A**) Colony morphology of *F. oxysporum* strains FCR51 and FCR24. (**B**) Agarose gel electrophoresis of the dsRNA-enriched extract obtained by cellulose column chromatography. (**C**) Schematic representation of the FoPV1 genome. Box and single dotted lines indicated the open reading frames (ORFs) and coding loci, respectively.

The *F. oxysporum* strains FCR51 and FCR24 were isolated from the root of an *S. miltiorrhiza* dry rot plant in Fangcheng City, Henan Province, China (Figure 1A). Two double-stranded RNA (dsRNA) segments (named dsRNA1 and dsRNA2) were extracted from strains FCR51 and FCR24, and they had estimated sizes of about 1.7 Kb and 1.5 Kb, respectively, after treatment with S1 Nuclease and DNase I (Figure 1B). The virus-free *F. oxysporum* strain DS42-2 was used as a control.

### 3.2. Molecular Characterization of FoPV1 in Strain FCR51

For the follow-up study, the *F. oxysporum* strain FCR51 was selected, and the sequence information of two dsRNA segments was obtained by sequence cloning and sequencing. The result showed that dsRNA1 (GenBank Accession No. OQ418724) was 1773 nucleotides (nt) in length with a GC content of 51.1%, and dsRNA2 (GenBank Accession No. OQ418725) was 1570 nt long with a GC content of 50.3% (Figure 1C). Sequencing analysis indicated that dsRNA1 contained one ORF from nt 63 to 1682, which putatively encodes a protein with 539 amino acid (aa) residues and a molecular mass of 62.6 kDa from an AUG triplet to a UAA triplet; the dsRNA2 also contained one ORF that initiated at position 123 and terminated at 1415, encoding 430 aa residues with a molecular mass of 46.6 kDa. (Figure 1C).

After conducting a homology search on BLASTP, it was found that the dsRNA1 protein product shares closely significant similarities with the RdRps of several partitivirus including Fusarium mangiferae partitivirus 2 (94.06% aa sequence identities, GenBank Acc No. UBZ25878.1), Phomopsis asparagi partitivirus 1 (87.94% aa sequence identities, GenBank Acc No. WEC89325.1), and Metarhizium brunneum partitivirus 2 (87.01% aa sequence identities, GenBank Acc No. QTC11257.1) (Table 1). Meanwhile, the putative protein encoded by dsRNA2 also shares certain similarities with the CP of partitivirus like Fusarium mangiferae partitivirus 2 (82.07% aa sequence identities, GenBank Acc No. UBZ25879.1), Metarhizium brunneum partitivirus 2 (70.3% aa sequence identities, GenBank Acc No. QTC11258.1), Colletotrichum partitivirus 1 (71.86% aa sequence identities, GenBank Acc No. ALD89089.1), etc. (Table 2).

**Figure 2:**
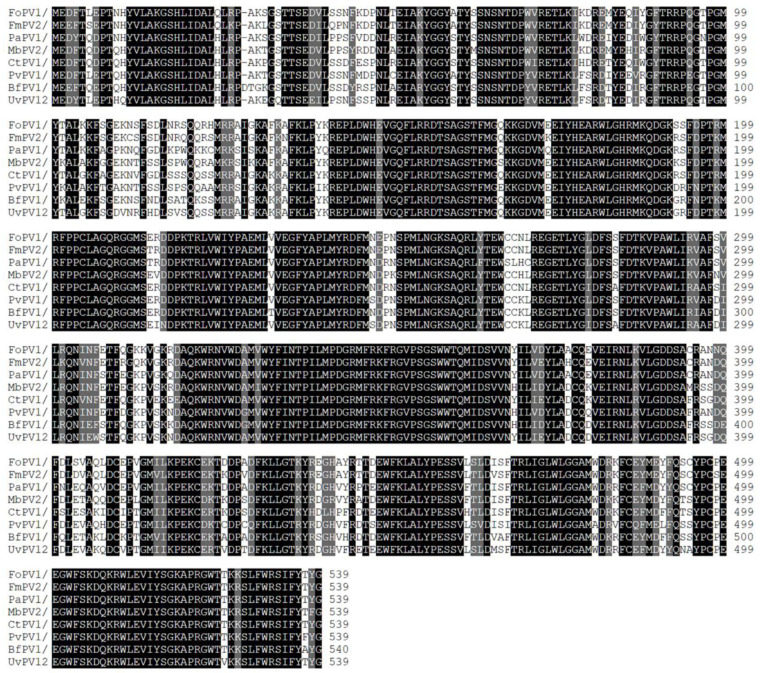
Multiple alignment of the conserved RdRp amino acid motifs encoded by FoPV1 and other *Partitiviridae* family members.

For further verification of the sequence characterization and comparability of FoPV1 with other partitiviruses, a conserved domain database search and multiple protein alignment were used. The result suggested that highly conserved amino acid motifs were present in and characteristic of the RdRp regions of FoPV1 with other members of family *Partitiviridae* (Figure 2). The CP regions of FoPV1 and other partitiviruses also contained a few conserved amino acid motifs (Figure S1).

The above results showed that the virus isolated from *F. oxysporum* strain FCR51, contained two dsRNA segments, and it was a new member of *Partitiviridae* family. Thus, we designated it Fusarium oxysporum partitivirus 1 (FoPV1).

### 3.3. Phylogenetic Analysis of FoPV1

**Figure 3:**
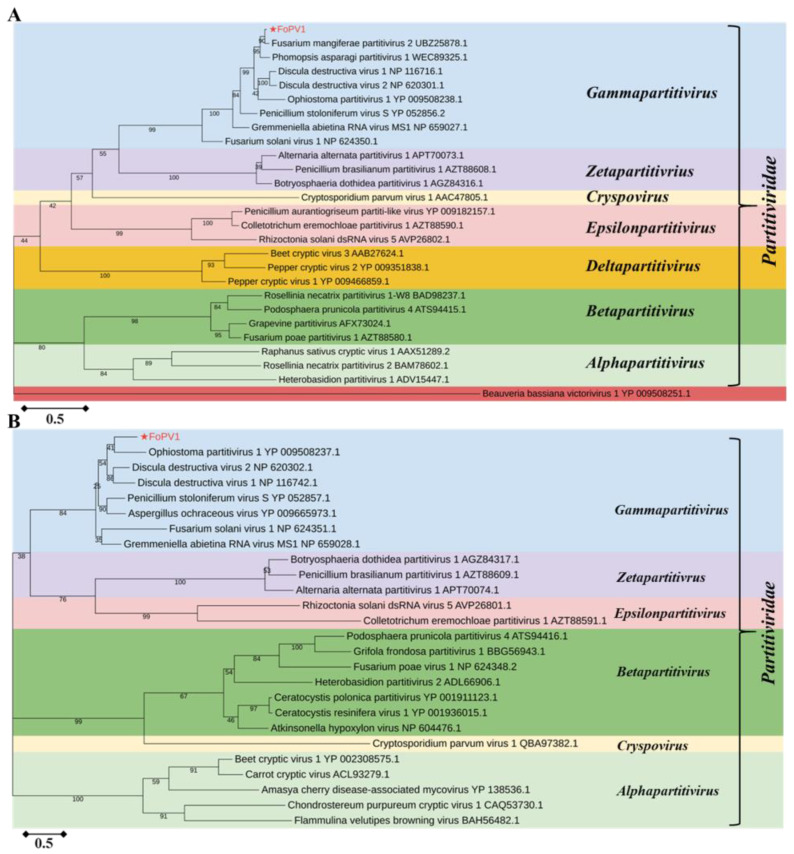
Phylogenetic analysis of FoPV1 with other related viruses. (**A**) Maximum likelihood (ML) phylogenetic tree of RdRp of FoPV1, *Partitiviridae* family members, and an outgroup virus. (**B**) Maximum likelihood (ML) phylogenetic tree based on the CP (ORF2) of FoPV1 and other members of the family *Partitiviridae*. The phylogenetic analyses were generated with the software MEGA11 using 1000 bootstrap replicates.

To examine the taxonomic classification and the phylogenetic relationship of FoPV1, the RdRP (ORF1) protein sequences of the FoPV1 and proteins with representative members from the genera *Gammapartitivirus*, *Zetapartitivirus*, *Cryspovirus*, *Epsilonpartitivirus*, *Deltapartitivirus*, *Betapartitivirus*, and *Alphapartitivirus* were aligned. Then, the phylogenetic tree was constructed with a maximum likelihood (ML) method (1000 bootstrap replicates). The result suggested that FoPV1 was one new member of the genera *Gammapartitivirus* belonging to the family *Partitiviridae* (Figure 3A). The phylogenetic trees based on the CP (ORF2) protein sequences between FoPV1 and other *Partitiviridae* family members also supported the same result (Figure 3B).

### 3.4. Effects of FoPV1 on F. oxysporum Strain FCR51

#### 3.4.1. Biological Effects of FoPV1 on *F. oxysporum* Strain FCR51

**Figure 4:**
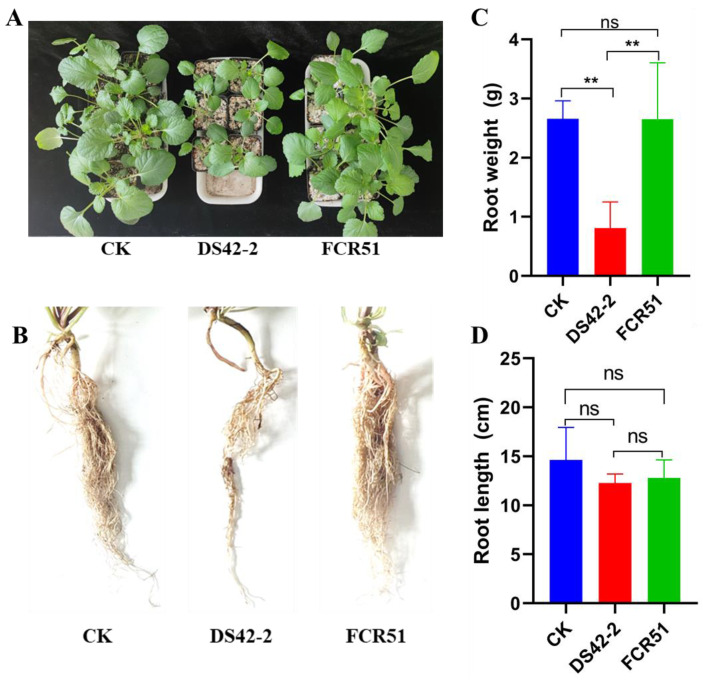
The biological effect detection in living *S. miltiorrhiza* seedlings. The growth status of stems and leaves (**A**) and roots (**B**) of *S. miltiorrhiza* 25 days after inoculation with 10 mL spores (10^7^ mL^−1^) of *F. oxysporum* strains FCR51 and DS42-2 (virus-free); (**C**,**D**) Effect of treatment with *F. oxysporum* strains FCR51 and DS42-2 on the roots’ weight and length. Non-treated plants served as controls. Double asterisks indicate highly significant differences (*p* < 0.01), and ns indicates no significant difference.

To determine the biological effect of the presence of FoPV1 in the *F. oxysporum* FCR51 strain, the strains FCR51 and DS42-2 (virus-free) were inoculated into *S. miltiorrhiza* seedlings at stage of development of the first two or three leaves; the pathogenicity and plant development data were investigated after 25 days, and non-inoculated plants served as controls (Figure 4). The results showed that the stems and roots of plants inoculated with strain FCR51 were grown conspicuously stronger than those inoculated with strain DS42-2 (Figure 4A,B). The root length has no clear distinction among different treatments, but the root weight inoculated with strain FCR51 was about three times as much as those inoculated with strain DS42-2 (Figure 4C,D, Table S2). Meanwhile, root rot symptoms were not evident in all treatments.

#### 3.4.2. Acquisition of FoPV1 Virus-Free Strain

To study the biological characteristics effects of FoPV1 infection to *F. oxysporum*, the strain FCR51 was cured by ribavirin treatment. The absence of the cured strain of FoPV1, named FCR51VF, was confirmed by RT-PCR analyses (Figure S2). This cured strain was used for studying the effect of FoPV1 on *F. oxysporum.*

#### 3.4.3. Effects of FoPV1 on *F. oxysporum* Strain FCR51

**Figure 5:**
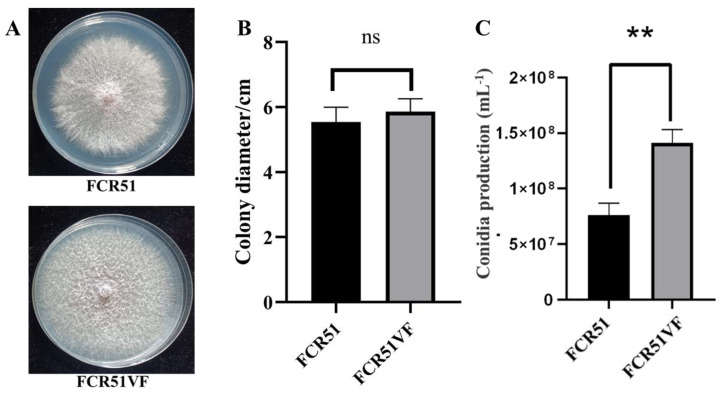
Characterization of FoPV1-infected and cured *F. oxysporum* strains FCR51 and FCR51VF. (**A**) Colony morphology of strains FCR51 and FCR51VF cultured on PDA medium for five days at 28 °C. (**B**) Colony diameters on a solid plate of strains FCR51 and FCR51VF. (**C**) Conidia production of strains FCR51 and FCR51VF quantified on PDB medium at 7 dpi. Double asterisks indicate highly significant differences (*p* < 0.01), and ns indicates no significant difference.

To identify the effects of FoPV1 on colony morphology, the strains FCR51 and FCR51VF were cultured on a PDA medium for five days. The colony edge of strain FCR51 appeared sparse and loose, whereas the strain was dense (Figure 5A). The colony diameters on solid plates were also measured for testing the growth differences of strains FCR51 and FCR51VF. The result indicated that the strain FCR51VF looked like a slightly bigger colony, and there was no significant difference in growth rates between the original and cured strains (Figure 5B).

The conidia production is important for the spread and virulence of *F. oxysporum*. Therefore, the ability to produce spores of strains FCR51 and FCR51VF were investigated after culturing on a PDB medium for a week. The result showed that the conidia production of the cured strain of FCR51VF was highly significantly increased compared to strain FCR51 (Figure 5C). Therefore, the infection of FoPV1 is correlated with colony edge morphology and reduced the spore production of *F. oxysporum*.

### 3.5. Influence of FoPV1 to the Virulence of F. oxysporum

**Figure 6:**
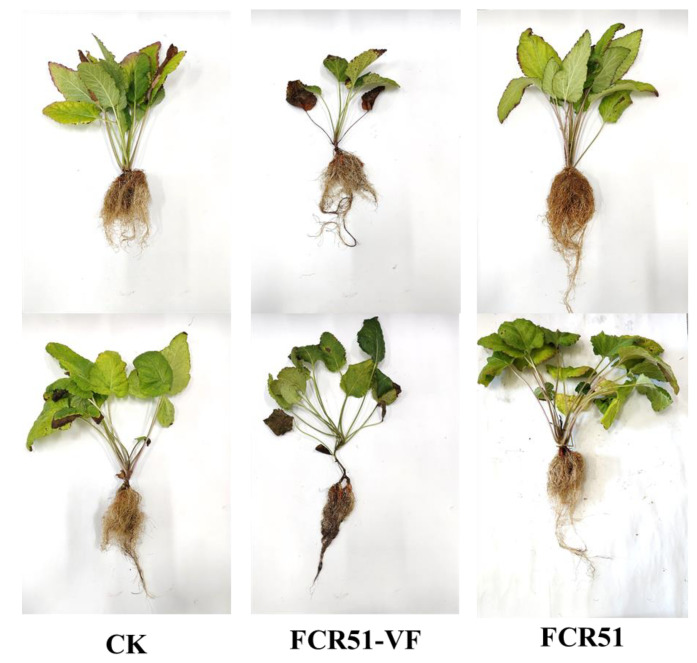
The pathogenicity detection in living *S. miltiorrhiza* seedlings. The symptoms of *S. miltiorrhiza* 25 days after inoculation with 10 mL spores (10^7^ mL^−1^) of *F. oxysporum* strains FCR51 and the cured strain of FCR51VF. Non-inoculated plants served as controls.

The root rot caused by *Fusarium* spp., including *F. oxysporum*, is a systemic infection disease that seriously threatens the safety of *S. miltiorrhiza* production. To determine whether the presence of FoPV1 reduced the virulence of *F. oxysporum* strain in terms of infecting *S. miltiorrhiza*, the strains FCR51 and FCR51VF were inoculated onto *S. miltiorrhiza* seedlings at the stage of development of the first two or three leaves, and the pathogenicity data were investigated 25 days after inoculation. Non-inoculated plants served as controls (Figure 6). The basal leaves of *S. miltiorrhiza* inoculated with the FCR51VF strain showed vein wilting, and the roots appeared brown or even black, especially the base roots. Conversely, there were no obvious symptoms on the leaves or roots of *S. miltiorrhiza* inoculated with the cured strain of FCR51 (Figure 6). Moreover, the roots and overground parts of the plants have appeared more stronger inoculated with the cured strain of FCR51 than those inoculated with the cured strain of FCR51VF (Figure 6).

## 4. Discussion

Over the past few years, there has been a dramatic increase in the generation of mycovirome sequences with low cost next-generation sequencing [2,44,45]. Numerous mycoviruses have been described as phytopathogenic fungi, entomopathogenic fungi, and even biological control fungi [2,3,46]. So far, serval mycoviruses have been identified in *F. oxysporum*, an important phytopathogenic species [35,36,37], but no report of any mycovirus belonging to the family *Partitiviridae*. In this study, we reported a novel mycovirus, FoPV1, from the *F. oxysporum* strain FCR51, belonging to the family of *Partitiviridae*.

A mass of partitiviruses have been found from a wide variety of phytopathogenic fungi, oomycetes, biological control fungi, and even human pathogenic fungi [8,9,10,11,12,13,14,15,16,17,18,19,20,21]. Partitiviruses generally have a typical genome composed of two major dsRNA segments and each one contains a single ORF encoding RdRP or CP, respectively [18,47]. Currently, viruses in the *Partitiviridae* family are divided into five recognized genera: *Alphapartitivirus*, *Betapartitivirus*, *Cryspovirus*, *Deltapartitivirus*, *Gammapartitivirus*, and two proposed genera: *Epsilonpartitivirus*, *Zetapartitivirus* [6,18]. FoPV1, reported here, has features typical of partitivirus, with a genome composed of two dsRNA segments (dsRNA 1 and dsRNA 2), encoding the RdRp and CP proteins, respectively. The homology blast and multiple protein alignment showed high pairwise-identity scores of the RdRp and CP of FoPV1 to other known partitivirus. In addition, phylogenetic analysis based on the RdRp and CP sequences also indicated that FoPV1 was clustered with members of the genus *Gammapartitivirus* in the family *Partitiviridae*. Therefore, FoPV1 was a new member of the genus *Gammapartitivirus* in the family *Partitiviridae*. To the best of our knowledge, this is the first report of a partitivirus from the pathogenic fungus *F. oxysporum*.

Some mycoviruses have been found to reduce the virulence of their fungal hosts causing disease in plants [1,23]. This property, known as hypovirulence, has been extensively identified in a large number of mycoviruses belonging to different families, like the Botrytis cinerea debilitation-related virus (BcDRV), which can reduce the virulence of *B. cinerea*, Rhizoctonia solani partitivirus 2 (RsPV2), which confers hypovirulence for *R. solani*, and so on [24,25,26]. Most mycovirus of the family *Partitiviridae* are generally latent on their hosts [8,9], and one mycovirus (TmPV1) can enhance the virulence of its fungal host [21]. Additionally, some partitiviruses can induce host hypovirulence itself, such as SsPV1, ClPV1, BcPV2, CaPV1, etc. [16,17,18,27]. Moreover, a few partitiviruses could affect the virulence of their fungal hosts by co-infecting with other mycoviruses [20,28], while there is no report of hypovirulence caused by a partitivirus in the pathogenic fungus *F. oxysporum*. In this study, FoPV1 was shown to have a reduced pathogenicity phenotype according to the comparison of the virulence of the present strain of FoPV1 (FCR51) and the cured strain of the virus (FCR51VF). Moreover, compared with the cured strain (FCR51VF), FCR51 has a lower conidia production, which is a positive correlation with the virulence of *F. oxysporum* [48]. Thus, FoPV1 has obvious hypovirulence to *F. oxysporum* by directly or indirectly reducing sporulation function.

*F. oxysporum* is generally recognized as an important pathogenic fungus because of causing vascular wilt of crop species [29]. But beyond that, *F. oxysporum* can also infect many plants roots, leading to root rot disease [30,31]. The root rot caused by *Fusarium* spp. including *F. oxysporum* could seriously threaten the safety of *S. miltiorrhiza* production. Using hypovirulent mycoviruses to control fungal diseases provides a new scheme for green prevention and the control of plant diseases; for example, CHV1 has been successfully used to control chestnut blight in Europe [32], and SsHADV1 is used to control disease caused by *S. sclerotiorum* [33,34]. As far as know, three hypovirulent mycoviruses have been reported in *F. oxysporum* [35,36,37]. FodV1 can decrease the colonizing efficiency of its fungal host [36]; moreover, both FoOuLV1 and FoAV1 have good biocontrol characteristic of *Fusarium* wilt which affects bitter melon and cucumber [35,37]. However, they were all explored as potential biocontrol agents against *Fusarium* wilt, and no hypervirulent mycovirus was reported for *F. oxysporum* causing root rot. The FoPV1 appeared to have a good biocontrol effect against root rot of *S. miltiorrhiza.* Intriguingly, the strain FCR51 that carries the virus FoPV1 could promote the growth of plants according to pot experiment analysis. In conclusion, a novel hypovirulent partitivirus (FoPV1) may become a new biocontrol agent for controlling the root rot of *S. miltiorrhiza.*

In this study, we characterized a novel mycovirus (FoPV1) related to members of the genus *Gammapartitivirus* in the family *Partitiviridae* from a phytopathogenic fungus, *F. oxysporum*. This is the first report of a partitivirus in *F. oxysporum*. The FoPV1 infection can cause hypovirulence to *F. oxysporum*, and it is also a potential biocontrol agent that could be further studied for controlling the root rot of *S. miltiorrhiza*.

## Figures and Tables

**Table 1:** Identities of RdRp of FoPV1 and those of partitivirus mycoviruses.

Virus Name	Accession	Query Cover	Per. Ident	E Value
Fusarium mangiferae partitivirus 2	UBZ25878.1	99%	94.06	0
Phomopsis asparagi partitivirus 1	WEC89325.1	99%	87.94	0
Metarhizium brunneum partitivirus 2	QTC11257.1	99%	87.01	0
Colletotrichum truncatum partitivirus 1	ALF46547.1	99%	87.01	0
Plasmopara viticola lesion associated Partitivirus 1	QHD64804.1	99%	86.64	0
Botryotinia fuckeliana partitivirus 1	YP_001686789.1	99%	84.63	0
Ustilaginoidea virens partitivirus 12	UVX28928.1	99%	84.79	0

**Table 2:** Identities of CP of FoPV1 and those of partitivirus mycoviruses.

Virus Name	Accession	Query Cover	Per. Ident	E Value
Fusarium mangiferae partitivirus 2	UBZ25879.1	91%	82.07	0
Metarhizium brunneum partitivirus 2	QTC11258.1	99%	70.3	0
Colletotrichum partitivirus 1	ALD89089.1	99%	71.86	0
Sodiomyces alkalinus partitivirus 2	ATP85066.1	99%	68.14	0
Beauveria bassiana partitivirus 2	CUS18594.1	99%	69.28	0
Phomopsis asparagi partitivirus 1	WEC89326.1	99%	68.14	0
Magnaporthe grisea partitivirus 1	AZT88597.1	99%	65.58	0

## Data Availability

Data are contained within the article and Supplementary Materials.

## Supplementary Materials

The following supporting information can be downloaded at: https://www.mdpi.com/article/10.3390/jof10030179/s1, Figure S1: Multiple alignment of the conserved CP amino acid motifs encoded by FoPV1 and other Partitiviridae family members; Figure S2: Amplification the RdRp sequence of FoPV1 by RT-PCR in strains FCR51and FCR51VF; Table S1: A list of primers used in this study; Table S2: Identity between CP of FoPV1 and those of alternavirus mycoviruses.
