# Supplementary material for: Molecular Characterization of the First Partitivirus from a Causal Agent of Salvia miltiorrhiza Dry Rot
Source: J Fungi (Basel). 2024 Feb 27;10(3):179. doi: 10.3390/jof10030179 (PMC10971513; doi:10.3390/jof10030179)
Supplement: Supplementary file 1 [file jof-10-00179-s001.zip › jof-2883716-supplementary.pdf]

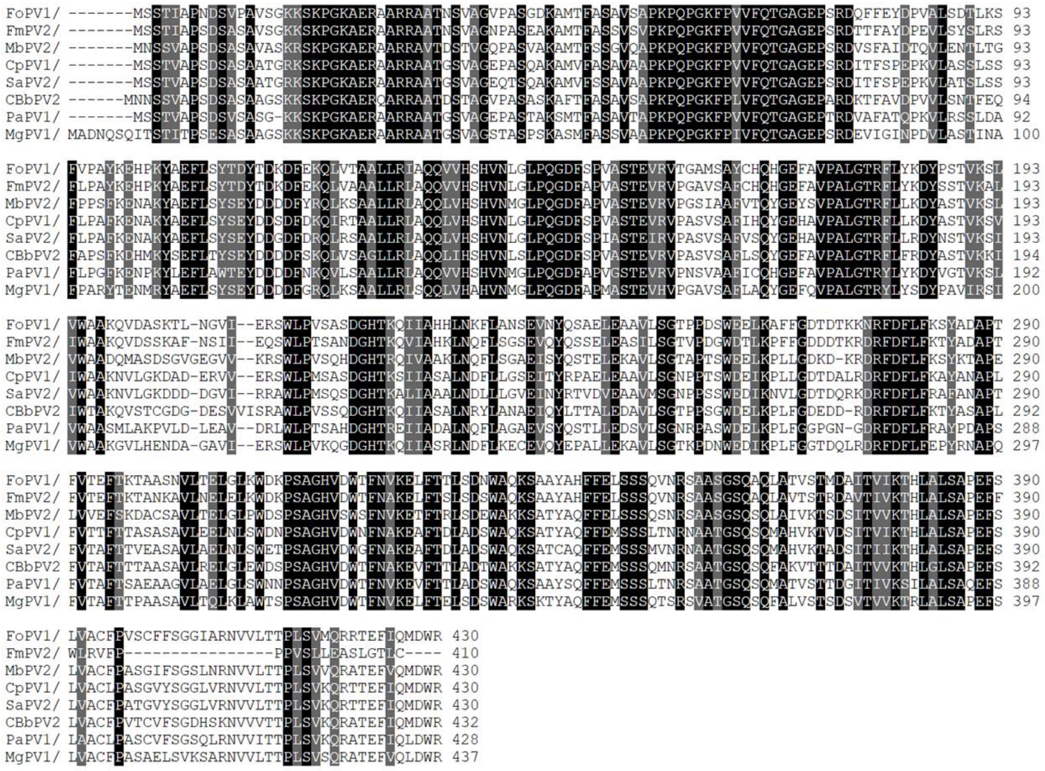

Figure S1. Multiple alignment of the conserved CP amino acid motifs encoded by FoPV1 and other *Partitiviridae* family members

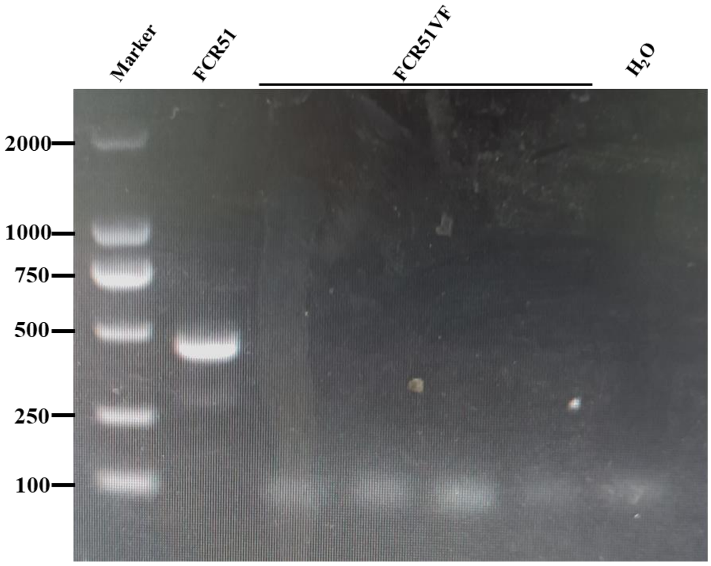

Figure S2. Amplification the RdRp sequence of FoPV1 by RT-PCR in strains FCR51and FCR51VF.

Table S1. A list of primers used in this study.

Primer Name	Oligonucleotide sequence (5′- 3′)	Usage
RACE3RT	CGATCGATCATGATGCAATGCNNNNNN	For initial sequence cloning
RACE3	CGATCGATCATGATGCAATGC	For terminal sequence cloning
pC2	CCGAATTCCCGGGATCC	
pC3-T7loop	p-GGATCCCGGGAATTCGGTAA TACGACTCACTATATTTTTATAGT GAGTCGTATTA-OH	
FCR51-rdrp-3'-long	ACTGTGAACCTGTCGGAATGAT	
FCR51-rdrp-3'-short	GCTTGCTCTCTATCCCGAATCT	
FCR51-rdrp-5'-long	AAACTGTCCGACTTCGTGCC	
FCR51-rdrp-5'-short	TCTCGAACCCAAGGATCAGTGT	
FCR51-cp-5'-short	GGCTTAGATTTCTTTCCCGACA	
FCR51-cp-5'-long	AGTCCTTATCGGTATAGTCGGTGTAG	
FCR51-cp-3'-long	TGTGGGCAGCAAAACAAGTAG	
FCR51-cp-3'-short	AACCTTCGTCACCGAGTTTACC	
FCR51-rdrp-3'-long	ACTGTGAACCTGTCGGAATGAT	
FCR51-1F	TTGTTGAGGGATTCTACGCC	Specific primers of the dsRNA1 segment of FoPV1
FCR51-1R	TCTACTTCTTGACAGGCGGC	
FCR51-2-F	GGTAAGAGTCACTGGGGCAATG	Specific primers of the dsRNA2 segment of FoPV1
FCR51-2-R	TAGAAGCCACGGGAGAAAAATC	

Table S2. Identity between CP of FoPV1 and those of alternavirus mycoviruses.

	**CK**	**DS42-2**	**FCR51**
**Root length (cm)**	14.63±3.316	12.28±0.923	12.8±1.836
**Root weight (g)**	2.659±0.3047	0.8088±0.4432	2.651±0.9521
